# Long-term trends of pH, alkalinity, and hydrogen ion concentration in an upwelling-dominated coastal ecosystem: Ría de Vigo, NW Spain

**DOI:** 10.1038/s41598-024-68694-z

**Published:** 2024-08-02

**Authors:** Sara Cameselle, Antón Velo, María Dolores Doval, Daniel Broullón, Fiz F. Pérez

**Affiliations:** 1 Instituto de Investigaciones Marinas, CSIC, Eduardo Cabello 6, 36208 Vigo, Spain; 2 Facultad de Ciencias del Mar, Universidade de Vigo, 36310 Vigo, Spain; 3 Instituto Tecnológico para el Control del Medio Marino de Galicia (INTECMAR), Peirao de Vilaxoan, 36611 Vilagarcía de Arousa, Spain

**Keywords:** Marine chemistry, Marine chemistry, Marine chemistry

## Abstract

The present study focuses on the Ría de Vigo (NW Spain), a coastal embayment influenced by the Canary Current Upwelling System, which is among the world’s significant Eastern Boundary Upwelling Ecosystems. The research assesses historical changes in the marine carbonate system by generating 25-year weekly time series at six stations . Assessing ocean acidification in the region is complex due to diverse factors influencing coastal carbon dynamics, making predictions more challenging. To capture the specific dynamics in Ría de Vigo, ensembles of Neural Networks were applied. These networks were trained with a data set obtained in several oceanographic cruises, in order to retrieve pH, hydrogen ion concentration and alkalinity, achieving a root mean square error of 0.0272 pH units, 0.588 nmol $\hbox {kg}^{-1}$, and 10.6 $\upmu$mol $\hbox {kg}^{-1}$, respectively. Subsequently, time series of the selected variables were generated, applying data of predictors measured at the aforementioned stations . An increase in normalized alkalinity was observed for all stations, except in the surface layer at the innermost location. A decrease in pH and an increase in hydrogen ion concentration were observed for all points, with trends that exceed reported rates of ocean acidification in the open ocean.

## Introduction

Deforestation, land-use activities and emissions from fossil fuels, among others, increased the concentration of carbon dioxide from 277 parts per million (ppm) in 1750^1^, to 417 ppm in 2022^2^. Anthropogenic emissions disrupt a natural carbon cycle that has atmosphere, ocean and terrestrial biosphere as main reservoirs from sub-daily to millennial scales, being the ocean a mayor $\hbox {CO}_2$ sink, absorbing $26\%$ of total $\hbox {CO}_2$ emissions during last decade^3^.

Ocean $\hbox {CO}_2$ uptake alters seawater chemical speciation in a process known as ocean acidification. $\hbox {CO}_2$ reacts with water to form carbonic acid ($\hbox {H}_2$ $\hbox {CO}_3$), which then dissociates in bicarbonate ($\hbox {HCO}_3^-$) and carbonate ($\hbox {CO}_3^{2-}$), losing hydrogen ions in the process. This results in lower pH values and reduces the calcium carbonate saturation states^4^. There is a growing body of evidence of the impacts that acidification has on marine organisms. Despite the fact that calcifying organisms are the most affected, the impact on a diverse range of marine organisms is broader than previously thought^5^. Rates of ocean acidification vary among areas, depending on biogeochemical and physical processes. At the Atlantic Ocean there are values of $-0.0017 \pm 0.0001$ pH units per year at the Bermuda Atlantic Time-series Study (BATS), and of $-0.0018 \pm 0.0002$ pH units per year at the European Station for Time series in the Ocean at the Canary Islands (ESTOC). Similar rates occur at the Pacific Ocean, for instance, in Hawaii Ocean Time-series (HOT) there is a decrease of $-0.0016 \pm 0.0001$ pH units per year^6^.

Not only the pH, but also alkalinity (TA) is crucial to quantify ocean carbon dioxide uptake during times of global change. Alkalinity can be defined as the excess of hydrogen ion acceptors over donors, and plays a major role in ocean chemistry, in buffering and in calcium carbonate precipitation and dissolution^7^.

The present study is centered on the Ría de Vigo (NW Spain). It is one of the Galician Rías and, along with the adjacent continental shelf, an integral part of the northern boundary of the Canary Current Upwelling System. This system is recognized as one of the most prominent Eastern Boundary Upwelling Ecosystems globally^8^. In the summer season, predominant northerly winds drive the movement of surface water away from the coast, facilitating the upwelling of deeper, cooler, nutrient-rich waters. This upwelling process results in a nourishing impact on the coastal waters, providing essential support to biodiversity^9^. As a coastal zone, processes related to the carbon system are expected to be different from those at the open ocean. Coastal zones are places where land-dominated and ocean-dominated processes coalesce. Hydrological regimes and horizontal flows sustain mechanisms for energy gradients and transfer of materials (nutrients, contaminants and sediments), which makes these areas more vulnerable to pressures resulting from human activities^10^. Consequently, the processes related to the carbon system in coastal areas are more dynamic and complex compared to those in the open ocean, resulting in larger pH ranges^6^.

To obtain time series of carbon system parameters, artificial neural networks (NN), a machine learning technique, have been applied in this study . Neural networks with at least one hidden layer have been proven to approximate nearly any function^11^. Some applications of machine learning in oceanography are: prediction of ocean weather and climate, habitat modelling and distribution, species identification, coastal water monitoring, marine resources management, detection of oil spill, pollution and wave modelling^12^.

While there are different architectures of NN, the simplest one is called feed forward network (FFN), which consists of neurons that are ordered into layers^13^. The application of FFN to the carbonate system has been widely used to predict variables such as alkalinity, as the work of^14^ to fill gaps in total alkalinity, and^15^ to understand the carbon system dynamics in an open ocean. Other studies were centred on yielding global data products, by which the user could obtain results from an already trained FFN, as the ones of^16^ and^17^. The approach of^16^ was lately improved by^18^ through an ensemble of Bayesian Neural Networks (BNN). Bayesian neural networks describe the parameters by a probability distribution instead of single values^19^. In the current study a similar approach is going to be taken.

Given the above, it is advisable to employ specially trained neural networks in coastal regions to capture the specific characteristics of the area in question, as global networks are designed for application in the open ocean. Thus, the necessity for a regional study in the area has motivated this research. The present study takes advantage of high quality data of different variables measured in the Ría de Vigo, to train an ensemble of NN in order to obtain time series of pH, total hydrogen ions ([$\hbox {H}^+$]), and alkalinity. This will allow for the assessment of ocean acidification in Ría de Vigo over the last decades.

## Methods

### Data

The data used in this study consists of two differentiated blocks: one to train the neural networks and other to make predictions. The database chosen for the training purpose is called ARIOS (Acidification in the Rías and the Iberian Continental Shelf)^20^. It was obtained from several oceanographic cruises conducted over four decades, from June 1976 to September 2018, carried out by the Instituto de Investigaciones Marinas (IIM), dependent of the Consejo Superior de Investigaciones Científicas (CSIC). ARIOS database is a compilation of biogeochemical properties, with discrete measurements of temperature, salinity, oxygen, nutrients, alkalinity, pH and chlorophyll. It was selected based on its reliability for long-term analysis. Oceanographic cruises were conducted along the Galician coast, with a special focus on the Ría de Vigo. This study selected data for training from the area between latitudes $\hbox {42}^{\circ }$ N and $\hbox {42.35}^{\circ }$ N, and the longitudes between $\hbox {8.6}^{\circ }$ W and $\hbox {9.11}^{\circ }$ W, in the upper 50 m of the water column (5755 data points for pH and 3850 for alkalinity). The temporal and data coverage is irregular, preventing the generation of continuous time series data. However, it provides sufficient data for neural networks to establish relationships between drivers, pH and alkalinity. A full description of ARIOS database can be found in^20^, where the sampling, analytical and quality control techniques are extensively described.

**Figure 1:**
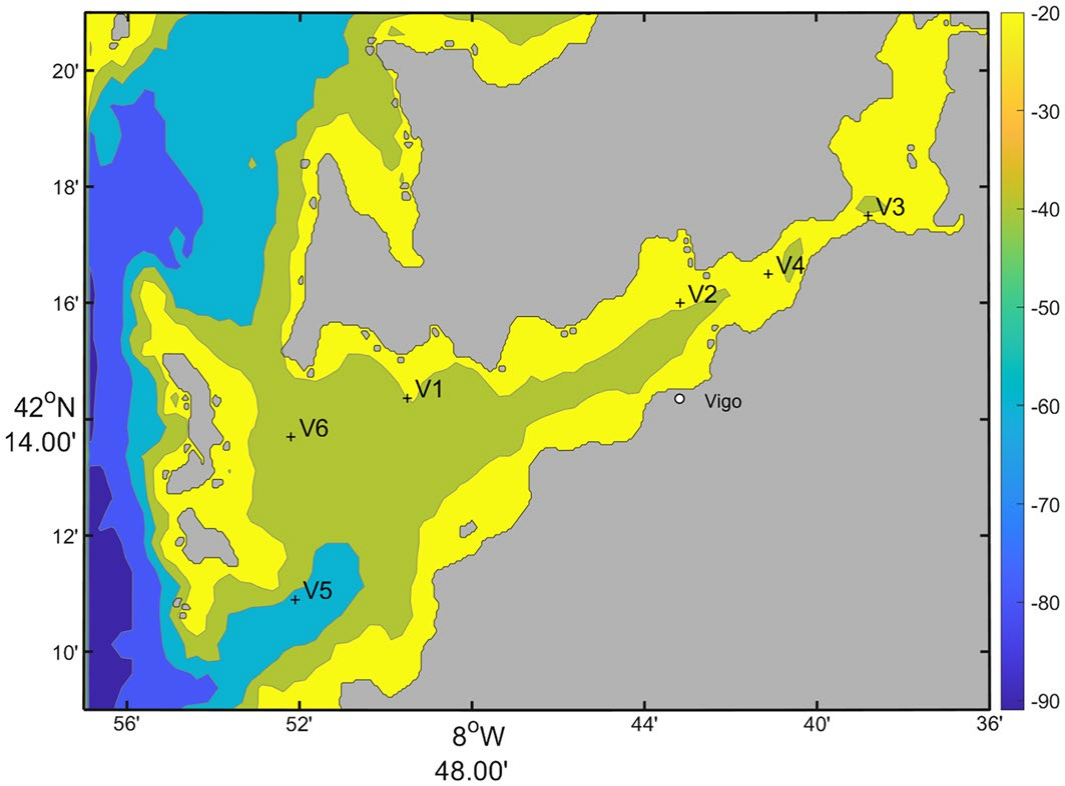
Map showing the six stations across Ría de Vigo (from V1 to V6). The color bar represents depth in meters.

The data used to feed the trained neural network, in order to obtain the outputs, comes from measurements by the Instituto Tecnolóxico para o Control do Medio Mariño (INTECMAR). Temperature, salinity, phosphate, nitrate, silicate and dissolved oxygen were measured on a weekly basis, in three depth ranges: 0–5 m, 5–10 m and 10–15 m. These variables were obtained in six stations across Ría de Vigo (Fig. 1) from 1995 to 2020. Further details of this database, including sampling techniques, can be consulted in^21^ and^22^.

### Neural networks

Alkalinity and pH are the target variables. The neural network architecture and the method used to obtain results, along with their errors, were similar for both . Thus, one ensemble of trained neural networks predicted alkalinity, and another one, with its particular weights, predicted the pH. During training, the weights were adjusted in order to obtain results as similar as possible to the target values. If this process continues without constraints, the model will adapt excessively to the peculiarities of the data, a phenomenon known as overfitting, thereby losing its generalization capability. To avoid this, the training data were randomly split into a training set $(90 \%)$ and a test set (the remaining $10 \%)$. The performance could be deduced from the test set, as it is independent and did not affect the training process.

The robustness of a neural network can be improved by combining the results of individual networks, in what is called a committee or ensemble model^17,18,23,24^. The first step involved creating each of the ten members that constitute the ensemble, followed by averaging their results to obtain the final output. In this case, the individual neural networks have the same architecture, since the process is stochastic, the results are expected to be slightly different, so averaging them will mitigate the error.

Each network is a multilayer perceptron of two hidden layers, with 28 neurons in the first layer and 10 in the second one for pH, and one hidden layer with 40 neurons for alkalinity. This combination was obtained after several trials were conducted in order to minimize the error. Bayesian regularization was used. Bayesian regularized neural networks are robust, they are difficult to overtrain or overfit, stopping training when necessary and effectively turning off weights that are not relevant^25^. The Matlab Neural Network Toolbox and the algorithm “trainbr” were chosen for this implementation.

To evaluate the performance of the model, the retrieved results were compared to the corresponding observations. Several statistical indices were used: mean squared error (MSE), root mean squared error (RMSE), mean absolute error (MAE) and the coefficient of determination ($r^2$). These statistics were performed on the test set in order to evaluate the ability of the model to generalize.

The chosen input variables for the networks were: latitude, longitude, depth, temperature, salinity, phosphate, nitrate, silicate, year and week. This decision was made based on the influence these variables have on the targets^26^. The periodicity of the input week was represented by its sine and cosine. Although in most cases the dissolved oxygen concentration mirrors the seasonal cycle of pH, oxygen was not chosen as an input given its low reliability in the INTECMAR database. Despite strong biological activity being the main driver of pH changes^20^, the other variables chosen were expected to account for it. This assumption is supported by precedents relaying in a different combination of inputs for predicting carbonate chemistry parameters, as^17^, requiring at least salinity and coordinate information.

### Long-term trends

The pH results were transformed into concentration of total hydrogen ions, in nanomoles per kg of seawater, that is determined to be:

1 $$\begin{aligned}{}[H^+] =10^{9-pH} \end{aligned}$$

Thus, pH obtained through the ensemble was transformed using Eq. 1, and then tested against the [$\hbox {H}^+$] test set values (previously transformed from pH). Notice that changes in pH represent a relative change in [$\hbox {H}^+$] rather than an absolute change^27^. This transformation was motivated due to evidence, suggesting that expressing acidification trends in [$\hbox {H}^+$] avoids the non-linearity of the logarithmic scale and because seawater $\hbox {pCO}_2$ has a considerably more linear $(99.5\%)$ relationship to [$\hbox {H}^+$] than to pH^28,29^.

Being salinity the main driver for alkalinity, its effect should be removed in order to analyse the underlying trend of alkalinity. The normalized total alkalinity (NTA) was calculated from TA using different methods to compare them and find the most appropriate one for this specific region. The simplest method is based in a reference salinity of 35, for which it was applied the following equation^30^:

2 $$\begin{aligned} NTA= TA \cdot \frac{35}{S} \end{aligned}$$

Where S is the salinity measured by INTECMAR for each specific value of projected TA. However, this traditional normalization concept has been criticised, since it is usually not able to adjust surface TA for salinity variations^31^. This is why^31^ propose the use of empirical relationships, as the following equation:

3 $$\begin{aligned} NTA= TA + \alpha \cdot ( 35 - S) \end{aligned}$$

Being $\alpha$ the slope of the linear regression of alkalinity data versus salinity. $\alpha$ was calculated for each station and depth, and for all data at the same time, as a global constant for the Ría de Vigo.

The outliers were determined to be values with a standard deviation greater than 3 units, and therefore were removed. Long-term trends were obtained for alkalinity, pH, and [$\hbox {H}^+$]. A seasonal detrending to remove the seasonality was applied for each variable and station. The method followed was the one applied by^32^, by which an oscillatory function is fitted to the data:

4 $$\begin{aligned} y(t)= A sin (\omega t + \phi ) + Bt + C \end{aligned}$$

Where $A sin (\omega t + \phi )$ is the seasonal component, and the parameter B corresponds to the trend of the data. Furthermore, after removing the seasonal component, a standard linear regression was performed to obtain the trend, i.e., B. The confidence intervals, $r^2$ and p-value were obtained from the linear regression.

## Results and discussion

### Neural networks performance

The statistics obtained from tests conducted on independent data are presented in Table 1. Each ensemble of NN provides lower errors (MAE, MSE and RMSE) and higher determination coefficients for the case of all variables, even better than the best individual NN. In the case of pH, while the best values for an individual NN are a RMSE of 0.030 and a $r^2$ of 0.89; the RMSE of the ensemble is 0.0272 pH units and the $r^2$ is 0.91. Same occurs in the case of TA with values of 10.6 $\upmu$mol $\hbox {kg}^{-1}$ and a $r^2$ of 0.97 from the ensemble. In the light of those results, it could be inferred that the ensemble provides the most accurate estimates. The same was found in other papers, making it a common approach in recent times, due to the power to smooth the weaknesses of individual NN^17,18,23,24^.

**Table 1:** Statistics of the ten NN and their ensemble for pH and TA, including the Mean Absolute Error (MAE), Mean Square Error (MSE), and Root Mean Square Error (RMSE).

Variable	NN	MAE	MSE	RMSE	r2
pH	1	0.0231	0.0011	0.0326	0.87
	2	0.0212	0.0009	0.0300	0.89
	3	0.0219	0.0010	0.0310	0.88
	4	0.0225	0.0010	0.0321	0.87
	5	0.0212	0.0009	0.0304	0.88
	6	0.0226	0.0010	0.0318	0.87
	7	0.0231	0.0011	0.0331	0.86
	8	0.0230	0.0010	0.0322	0.87
	9	0.0218	0.0009	0.0301	0.89
	10	0.0224	0.0010	0.0310	0.88
	**Ensemble**	**0.0189**	**0.0007**	**0.0272**	**0.91**
[$\hbox {H}^+$]	**Ensemble**	**0.399**	**0.346**	**0.588**	**0.91**
TA	1	7.3	133.2	11.5	0.96
	2	7.2	134.6	11.6	0.96
	3	7.3	145.0	12.0	0.96
	4	7.2	140.5	11.9	0.97
	5	7.2	168.0	13.0	0.95
	6	7.4	139.8	11.8	0.96
	7	6.9	125.9	11.2	0.96
	8	7.5	133.3	11.6	0.96
	9	7.8	158.6	12.6	0.96
	10	7.1	125.6	11.2	0.96
	**Ensemble**	**6.3**	**112.6**	**10.6**	**0.97**

Additionally, the table displays the [$\hbox {H}^+$] statistics derived from the pH ensemble results.

The statistics for [$\hbox {H}^+$] were not possible to compare with another papers, since there is a lack of studies obtaining it from NN. In the case of [$\hbox {H}^+$], the error depends on the pH NN, as this variable was transformed from pH applying Eq. 1. Therefore, the error of [$\hbox {H}^+$] is proportional to the one of pH, and so is the quality of the predictions.

The complexity in estuarine waters is higher than in the open ocean, which makes it more challenging for a NN to deduce the correlation between proxies and estimates. Processes as river-ocean mixing and upwelling influence aquatic acid-base properties in estuaries^33^. These conditions imply that the variation of the carbonate system in open sea is lower, and therefore the error rates obtained from NN. An example of open sea predictions for pH is feed forward NN CANYON with RMSE of 0.019^16^, improved by Bayesian NN CANYON-B with a value of 0.013^18^. The Bayesian NN CANYON-MED retrieves a RMSE of 0.016 and a $r^2$ of 0.86^23^ for the Mediterranean Sea, which as a semi-enclosed marginal sea has its own particularities, although is not as heterogenous in a short temporal and spatial scale as an estuary. Regarding TA, in open sea the values obtained for the RMSE are 7^16^, a range of 3–6.2^34^, and 6.3 $\upmu$mol $\hbox {kg}^{-1}$^18^.

There is a lack of studies applying NN to coastal ecosystems, as cited below. In the case of^35^ the MAE is 0.00099,^15^ predicts pH with a RMSE of 0.03 and^32^ yields a 0.64 error using a recurrent NN. Regarding TA,^15^ predicted it with RMSE of 6.7 and a $r^2$ of 0.95, and^23^ obtains a RMSE of 13 $\upmu$mol $\hbox {kg}^{-1}$. The errors of the present paper fall within the same range, which supports the effectiveness of the current NN model for an accurate prediction of the target variables in the Ría de Vigo.

### Time series

**Figure 2:**
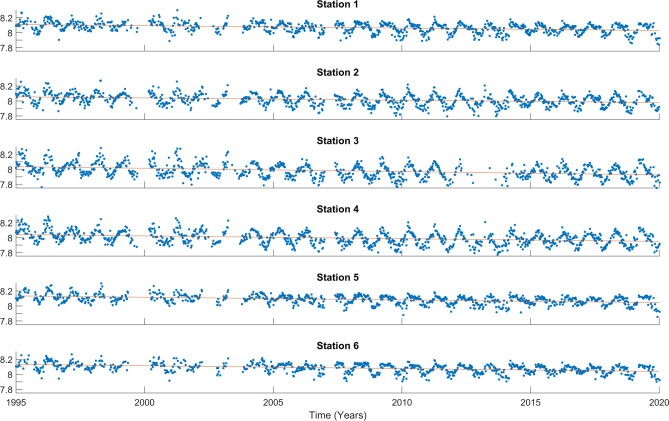
The pH time series (blue dots) are displayed for the 0–5 m range at the 6 stations. The trend is depicted by a red line. The y-axis represents pH in pH units, and the x-axis represents the years.

**Figure 3:**
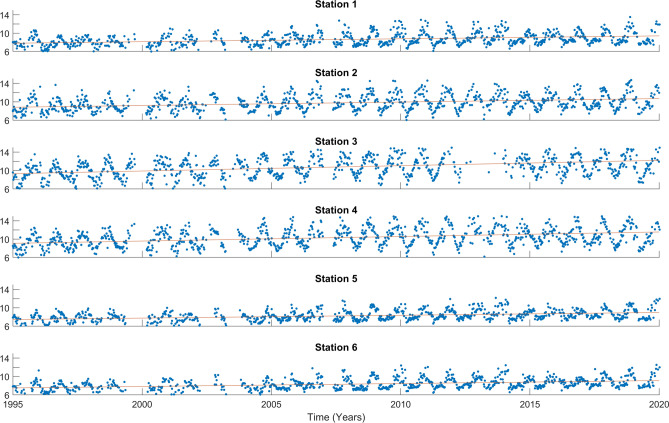
The [$\hbox {H}^+$] time series (blue dots) within the 0–5 m range at the 6 stations, with a trend indicated by a red line. The y-axis represents [$\hbox {H}^+$] in nmol $\hbox {kg}^{-1}$, and the x-axis represents the years.

The time series of pH and [$\hbox {H}^+$] are shown in Figs. 2 and 3, respectively, for the surface. For the other depths, they are presented in Supplementary Figs. S1, S2, S3, and S4. The long-term trends are shown in Table 2. All results are statistically significant. The acidification trends at Ría de Vigo range between $-$ 0.0025 and $-$ 0.0042 pH units per year through the time span covered by this study. The values found at the open ocean are $-$ 0.0017 (BATS), $-$ 0.0018 (ESTOC) and $-$ 0.0016 (HOT) pH units per year^6^, and $-$ 0.0124 pH units per decade in the tropical area near the $\hbox {137}^{\circ }$ E meridian^36^. In the open ocean near the northwestern Iberian Peninsula, closely related to this study’s area, the trend is $-$ 0.0012 pH per year for the upper Central waters^20^. These open ocean trends are consistent with the globally averaged decrease of $-$ 0.0181 $\hbox {decade}^{-1}$^37^. Therefore, the rates of change are stronger and more variable at Ría de Vigo than in the open ocean, which is in agreement with the OSPAR assessment of Ocean Acidification in the North-East Atlantic^38^. According to the OSPAR assessment, trends are stronger towards the coast. Furthermore, Eastern Boundary Current Systems are expected to be one of the most affected coastal ecosystems by ocean acidification due to coastal upwelling^39,40^.

**Table 2:** Long-term trends of pH, [$\hbox {H}^+$], TA and NTA between 1995 and 2020

Station	Depth (m)	pH ($\hbox {yr}^{-1}$)	[$\hbox {H}^+$] (nmol $\hbox {kg}^{-1}$ $\hbox {yr}^{-1}$)	TA ($\upmu$mol $\hbox {kg}^{-1}$ $\hbox {yr}^{-1}$)	NTA global ($\upmu$mol $\hbox {kg}^{-1}$ $\hbox {yr}^{-1}$)
V1	0–5	$-$0.0032± 0.0004	0.065 ± 0.008	1.40±0.47	0.60±0.22
	5–10	$-$0.0030±0.0005	0.061±0.008	1.07±0.32	0.57±0.15
	10–15	$-$0.0028±0.0004	0.059±0.009	0.65±0.26	0.39±0.12
V2	0–5	$-$0.0031±0.0004	0.070±0.010	0.88±0.66	0.03±0.33*
	5–10	$-$0.0028±0.0004	0.063±0.009	1.07±0.37	0.56±0.19
	10–15	$-$0.0025±0.0004	0.063±0.012	0.69±0.30	0.46±0.15
V3	0–5	$-$0.0042±0.0005	0.103±0.011	$-$0.24±1.39*	$-$2.52±0.85
	5–10	$-$0.0034±0.0004	0.085±0.010	0.94±0.54	0.39±0.31*
	10–15	$-$0.0032±0.0004	0.081±0.009	0.87±0.43	0.56±0.25
V4	0–5	$-$0.0037±0.0004	0.085±0.010	0.40±0.82*	$-$0.60±0.49*
	5–10	$-$0.0030±0.0004	0.071±0.009	0.82±0.41	0.57±0.21
	10–15	$-$0.0028±0.0004	0.072±0.014	0.65±0.33	0.43±0.17
V5	0–5	$-$0.0035±0.0004	0.065±0.007	1.66±0.41	0.47±0.17
	5–10	$-$0.0031±0.0003	0.060±0.007	1.18±0.29	0.51±0.12
	10–15	$-$0.0027±0.0004	0.053±0.007	0.57±0.22	0.38±0.10
V6	0–5	$-$0.0036±0.0004	0.070±0.008	1.56±0.50	0.23±0.27*
	5–10	$-$0.0031±0.0005	0.066±0.007	0.88±0.31	0.41±0.14
	10–15	$-$0.0028±0.0004	0.055±0.010	0.53±0.24	0.24±0.11

Values of NTA are calculated applying a global value of $\alpha$. Trends which are not statistically significant (*p*-value>0.01) are marked with an asterisk.

Regarding the spatial distribution of the trends, there is a clear pattern with depth. The surface layer (0–5 m) is the one with strongest acidification rates, which is true for all stations. This rate diminishes with depth, being lower at the deepest level analysed (10–15 m). These results agree with current observations, as for the North Atlantic water masses, the general pattern seems to be a decrease of the acidification rates over depth in all basins^41^. However, as mentioned before, coastal areas should be considered as having their own particularities. Examples of near-shore areas, as shown in^42^, also demonstrate a decrease in rate with depth within the first 20 meters.

Another pattern could be observed, as the trend of acidification in the most superficial layer (0–5 m) changes substantially between stations. Stations in the middle zone (V1 and V2) have the lowest rates. The inner station V3 has the highest rate, of $-$ 0.0042 pH units, which is more remarkable attending to the [$\hbox {H}^+$], because of the change of scale. However, the other layers (5–10 m, 10–15 m) seem not affected by the spatial distribution of the stations. The variability observed is due to the complex factors that influence the carbon dynamics in very near-shore waters, such as river run-off, land-ocean interactions, mixing dynamics and influence of benthic processes^10^.

The acidification process reduces the saturation state of aragonite ($\Omega _{arag}$) and calcite ($\Omega _{cal}$), the two primary mineral forms of calcium carbonate utilized by most bivalves for shell formation. Numerous shell-forming organisms have demonstrated high sensitivity to this phenomenon^43^. Given the rates observed in the current study, bivalves are going to be under considerable pressure if these trends endure. Hence, it is crucial to pay special attention to this circumstance, considering the importance of bivalves in the Ría de Vigo ecosystem and its economy, with particular emphasis on the economic significance of mussel farming using floating rafts.

**Figure 4:**
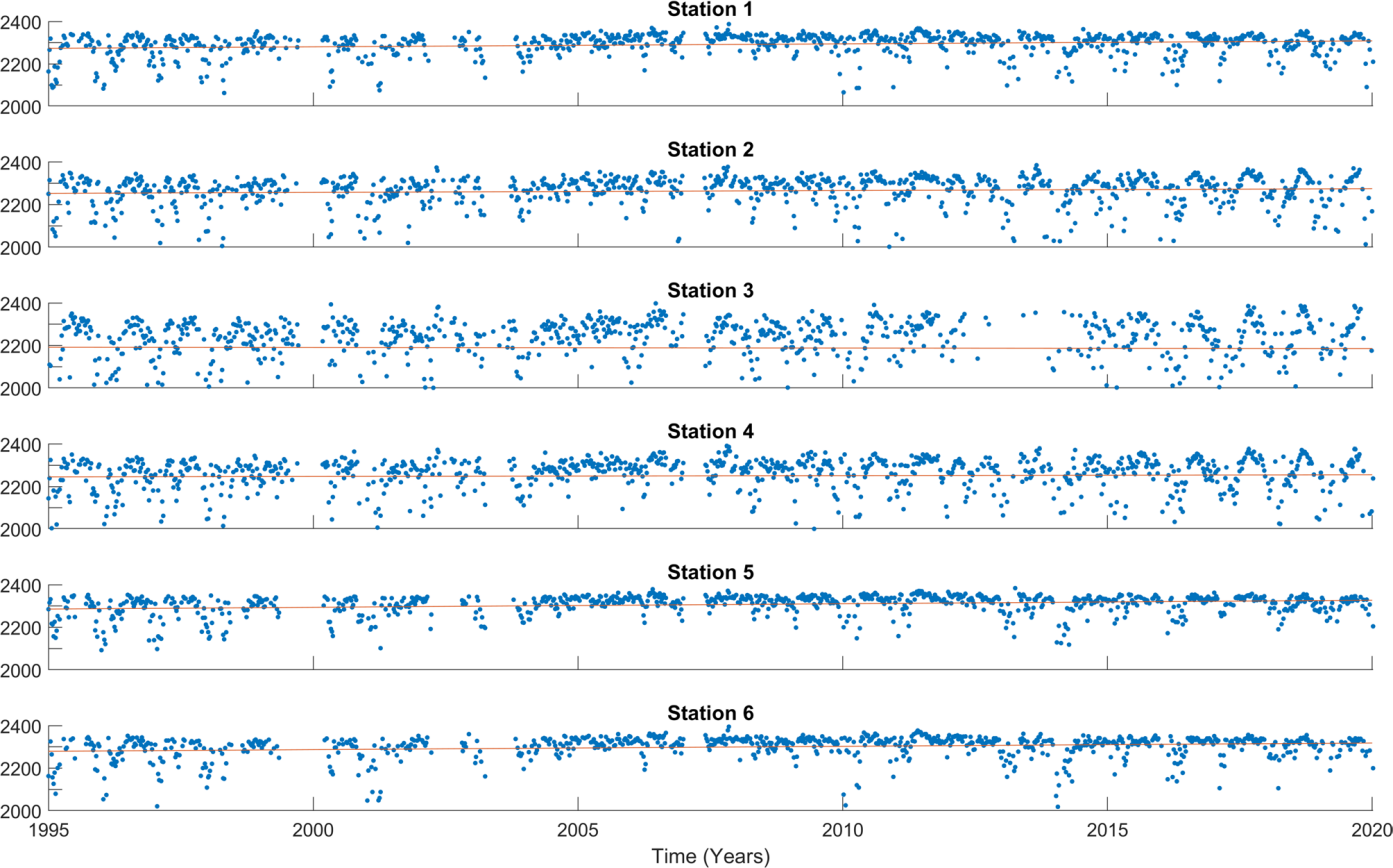
The TA time series (blue dots) are displayed for the 0–5 m range at the 6 stations. The trend is depicted by a red line. The y-axis represents TA in $\upmu$mol $\hbox {kg}^{-1}$, and the x-axis represents the years.

The time series of TA are shown in Fig. 4 for the surface, and in Supplementary Figs. S5 and S6 for the rest of depths. The long-term trends of TA and NTA are shown in Table 2. At all stations and depths, for data that is statistically significant, there is an increase in TA during the time span covered by this study. An increase in TA was also observed at the Mediterranean Sea with a value of 2.08 ± 0.19 $\upmu$mol $\hbox {kg}^{-1}$ $\hbox {yr}^{-1}$^44^.

Since salinity is the main driver of alkalinity, its effect should be removed to analyze the underlying pattern in the long-term alkalinity trend. The results of the three normalization methods are shown in Supplementary Table S1. Among these methods, only the one applying a global alpha was selected for Table 2. This decision was made based on the results of the relationship between alkalinity and salinity. The change in sign of the slope indicates over-correction by the traditional normalization scheme^31^. The global method was finally selected because of its convenience giving that the value of $\alpha$ does not change substantially between stations, being the global value 46.7 and a $r^2$ of 0.78. This makes possible to apply a unique value for the normalization of alkalinity at Ría de Vigo.

Data of NTA shows a homogeneous positive trend along the Ría and the different layers. However, the innermost station, V3, has a negative trend in the surface layer. The significantly lower $r^2$ of alkalinity versus salinity for this location compared to the others implies that alkalinity is mostly driven by other variables apart from salinity there. The station is near the river mouth and is shallower, making it more susceptible to seasonal changes and abrupt events. Although salinity is the most correlated predictor, it cannot explain the observed negative trend. The next most correlated predictor of alkalinity in the Ría de Vigo is silicate, according to the Pearson coefficient obtained from ARIOS^20^. The correlation is negative, and indeed, there is a strong positive trend in the measured silicate, which leads to a decrease in alkalinity.

The variability in alkalinity trends at the innermost station indicates that this areas might be less buffered against pH fluctuations, particularly under conditions of high river runoff. The reduction in buffering capacity means that additions of carbon dioxide lead to more pronounced decreases in pH, exacerbating the effects of ocean acidification. This could make local marine organisms more vulnerable to acidification, especially during events that lead to significant freshwater inputs^7^.

## Conclusion

To obtain the trends over the desired 25-year time frame, ensembles of neural networks have been successfully trained. These NN trained with ARIOS return low errors when applied to an independent dataset, supporting their ability to generalize in the studied area. Regarding the errors provided, the values of RMSE are as follows: 0.0272 pH units for pH, 0.588 nmol $\hbox {kg}^{-1}$ for [$\hbox {H}^+$] and 10.6 $\upmu$mol $\hbox {kg}^{-1}$ for alkalinity. These values are notably low, considering the high variability in coastal areas.

Over the course of the last 25 years, the marine carbonate system in the Ría de Vigo has undergone significant changes, evidenced by the accelerated rates of ocean acidification observed in this region. This study reveals a marked decline in pH levels, with rates ranging from $-$ 0.0025 to $-$ 0.0042 pH units per year, significantly surpassing those recorded in open ocean. This pattern is remarkably stronger when [$\hbox {H}^+$] is observed, due to the change of scale. Such robust acidification patterns are particularly pronounced in the surface waters (0–5 m), where the highest rates are consistently observed, decreasing with depth. Spatial variability is also evident, with the innermost stations exhibiting the most extreme acidification rates. Regarding alkalinity, it was normalized, since there is a strong relation with salinity. The best method is considered to be the one based on an empirical relationship, applying a global constant for all stations and depths. Alkalinity increased at all stations except in the surface layer of the innermost location, where a high increase in silicate was observed. The variability observed is due to the influence of local factors such as riverine input, land-sea interactions, and benthic processes^10, 45^ on the carbonate chemistry of the Ría de Vigo.

These findings highlight the unique challenges faced by the marine ecosystem in this area, especially for calcifying organisms like bivalves, which are crucial to both the ecological and economic fabric of the region. The substantial acidification observed poses a significant threat to these organisms, pressing the need for targeted research and conservation strategies to mitigate the impacts and preserve the marine biodiversity of the Ría de Vigo. Given that the economy of the Ría de Vigo heavily relies on mussel farming using floating rafts, understanding the estuary’s status in terms of acidification is crucial. This knowledge enables the implementation of coping strategies such as selective breeding and stock management, buffering systems, integrated multi-trophic aquaculture (IMTA), and habitat enhancement^46^. Additionally, awareness of the spatial variation in acidification levels within the estuary allows for more effective site selection. For instance, it is well-documented that seawater near river mouths tends to be more acidic compared to areas further from these inflows^47^, a situation also observed in the Ría de Vigo. Therefore, selecting sites away from river mouths can mitigate the impacts of low pH, optimizing the environment for mussel cultivation and ensuring the sustainability and productivity of aquaculture operations in the Ría de Vigo.

It would be pertinent for future investigations to extend their scope beyond pH, total alkalinity, and hydrogen ion concentration. Exploring additional variables within the carbon system, such as dissolved inorganic carbon (DIC), partial pressure of carbon dioxide ($\hbox {pCO}_2$), and carbonate ion concentration ([$\hbox {CO}_3^{2-}$]), could provide deeper insights into the complex dynamics of ocean acidification. Furthermore, integrating data on biological parameters, such as primary productivity, species composition, and calcification rates of marine organisms, would contribute to a more comprehensive understanding of the impacts of ocean acidification on marine ecosystems. By incorporating a multidisciplinary approach and considering a broader range of variables, future studies can advance our knowledge of ocean acidification processes and their ecological ramifications, ultimately informing more effective mitigation and adaptation strategies.

## Supplementary Information

Supplementary Information.

## Data Availability

The ARIOS dataset, employed for training the neural networks, is archived at Digital CSIC under the digital object identifier (DOI) (10.20350/digitalCSIC/12498). The input data from INTECMAR and the outcomes of the current analyses are publicly available on (https://zenodo.org/records/10392096).
