# Author Correction: Long-term trends of pH, alkalinity, and hydrogen ion concentration in an upwelling-dominated coastal ecosystem: Ría de Vigo, NW Spain

**DOI:** 10.1038/s41598-025-91517-8

**Published:** 2025-03-04

**Authors:** Sara Cameselle, Antón Velo, María Dolores Doval, Daniel Broullón, Fiz F. Pérez

**Affiliations:** 1 Instituto de Investigaciones Marinas, CSIC, Eduardo Cabello 6, 36208 Vigo, Spain; 2 Facultad de Ciencias del Mar, Universidade de Vigo, 36310 Vigo, Spain; 3 Instituto Tecnológico para el Control del Medio Marino de Galicia (INTECMAR), Peirao de Vilaxoan, 36611 Vilagarcía de Arousa, Spain

Correction to: *Scientific Reports* 10.1038/s41598-024-68694-z, published online 02 August 2024

The original version of this Article omitted an affiliation for author Sara Cameselle. The correct affiliations are listed below.

Instituto de Investigaciones Marinas, CSIC, Eduardo Cabello 6, 36208, Vigo, Spain

Facultad de Ciencias del Mar, Universidade de Vigo, 36310, Vigo, Spain

The original Article has been corrected.

