# Supplementary material for: Long-term trends of pH, alkalinity, and hydrogen ion concentration in an upwelling-dominated coastal ecosystem: Ría de Vigo, NW Spain
Source: Sci Rep. 2024 Aug 2;14:17929. doi: 10.1038/s41598-024-68694-z (PMC11297135; doi:10.1038/s41598-024-68694-z)
Supplement: Supplementary file 1 — Supplementary Information. [file 41598_2024_68694_MOESM1_ESM.pdf]

Long term trends of pH, total alkalinity and hydrogen ion concentration in the Ría de Vigo (NW Spain), an application of Artificial Neural Networks

Sara Cameselle[1,*], Antón Velo[1,*], María Dolores Doval[2], Daniel Broullón[1], and Fiz F. Pérez[1]

[1]Instituto de Investigaciones Marinas, CSIC, Eduardo Cabello 6, 36208 Vigo, Spain
[2]Instituto Tecnológico para el Control del Medio Marino de Galicia (INTECMAR), Peirao de Vilaxoan, 36611, Vilagarcía de Arousa, Spain
[*]scameselle@iim.csic.es
[*]avelo@iim.csic.es

SUPPLEMENTARY INFORMATION

Station	Depth (m)	TA	NTA	NTA global	NTA per station	alpha	r^2
V1	0-5	1.40±0.47	0.36±0.35*	0.60±0.22	0.64±0.21	43.3	0.79
	5-10	1.07±0.32	0.26±0.22*	0.57±0.15	0.56±0.15	42.9	0.80
	10-15	0.65±0.26	0.30±0.17	0.39±0.12	0.42±0.11	43.5	0.82
V2	0-5	0.88±0.66	-0.58±0.47*	0.03±0.33*	0.15±0.34*	43.2	0.73
	5-10	1.07±0.37	0.37±0.25	0.56±0.19	0.64±0.19	43.6	0.77
	10-15	0.69±0.30	0.29±0.18	0.46±0.15	0.46±0.15	45.7	0.80
V3	0-5	-0.24±1.39*	-5.35±2.00	-2.52±0.85	-2.23±0.85	41.0	0.56
	5-10	0.94±0.54	0.04±0.42*	0.39±0.31*	0.36±0.31*	49.0	0.73
	10-15	0.87±0.43	0.34±0.28*	0.56±0.25	0.48±0.25	49.2	0.76
V4	0-5	0.40±0.82*	-1.64±0.68	-0.60±0.49*	-0.62±0.50*	46.2	0.65
	5-10	0.82±0.41	0.37±0.28	0.57±0.21	0.59±0.21	45.7	0.77
	10-15	0.65±0.33	0.40±0.19	0.43±0.17	0.43±0.17	47.3	0.78
V5	0-5	1.66±0.41	-0.06±0.28*	0.47±0.17	0.60±0.16	41.2	0.81
	5-10	1.18±0.29	0.39±0.20	0.51±0.12	0.59±0.12	42.8	0.84
	10-15	0.57±0.22	0.31±0.15	0.38±0.10	0.39±0.10	42.1	0.81
V6	0-5	1.56±0.50	-0.22±0.39*	0.23±0.27*	0.39±0.26	40.3	0.78
	5-10	0.88±0.31	0.16±0.22*	0.41±0.14	0.47±0.13	41.9	0.82
	10-15	0.53±0.24	0.13±0.17*	0.24±0.11	0.28±0.11	39.7	0.79

Table S1. Long-term trends of TA in units of μmol kg^{-1} yr^{-1} applying different normalization methods. Trends which are not statistically significant (p-value<0.01) are marked with an asterisk. The value of r^2 shows the correlation between alkalinity and salinity. Values of alpha applied to the NTA per station are also shown.

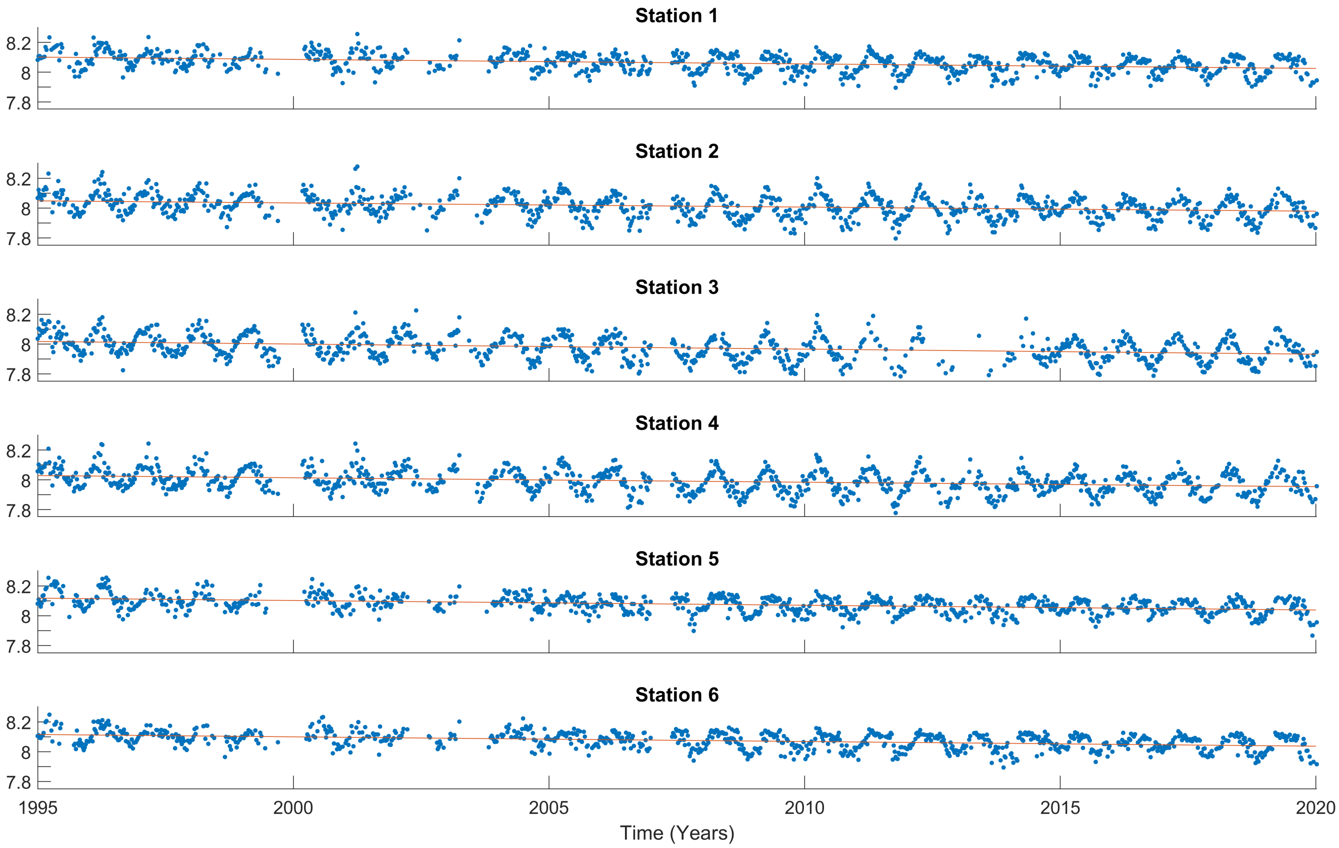

Figure S1. pH time series (blue dots) for the 5-10 m range at the 6 stations, with a trend indicated by a red line. The y-axis represents pH in pH units, and the x-axis represents the years.

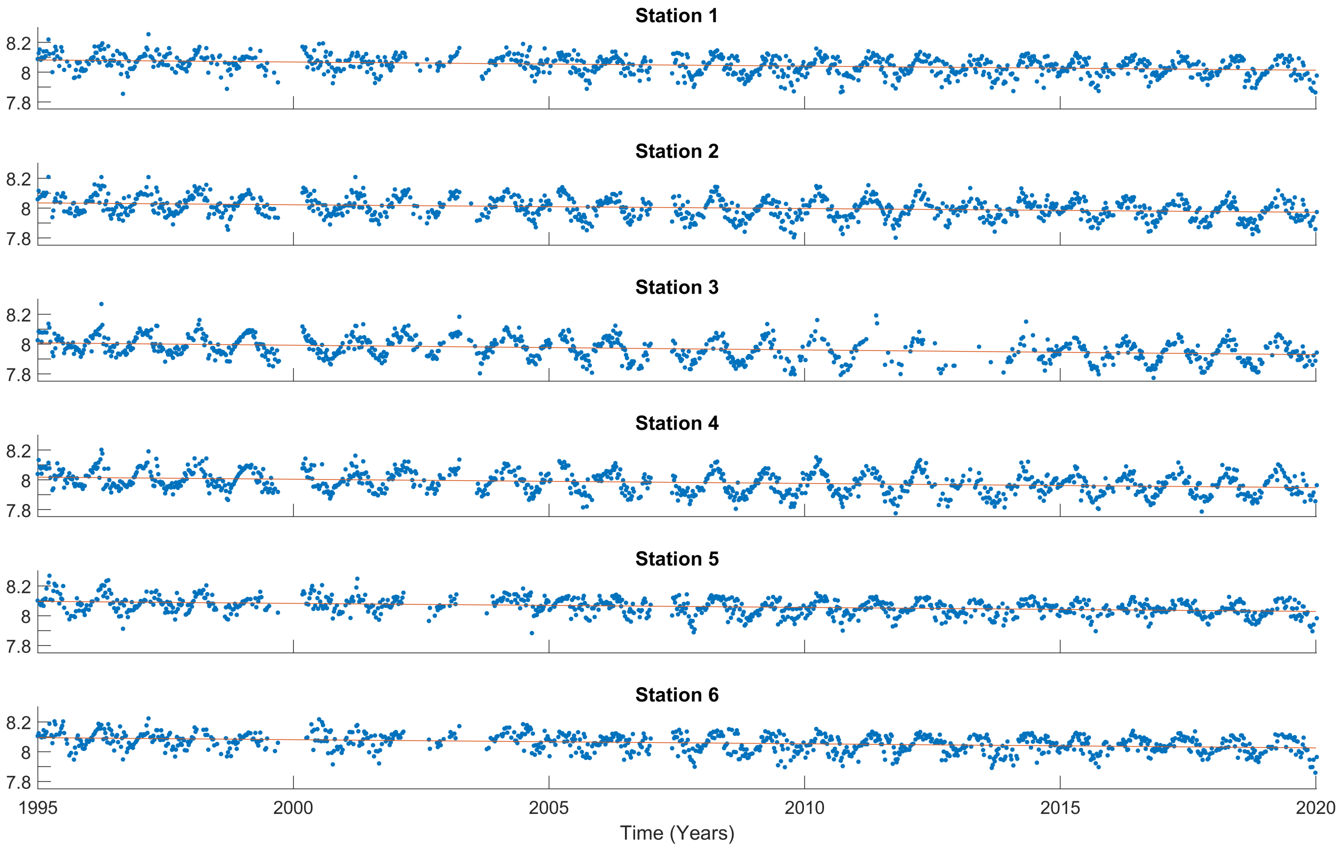

Figure S2. pH time series (blue dots) for the 10-15 m range at the 6 stations, with a trend indicated by a red line. The y-axis represents pH in pH units, and the x-axis represents the years.

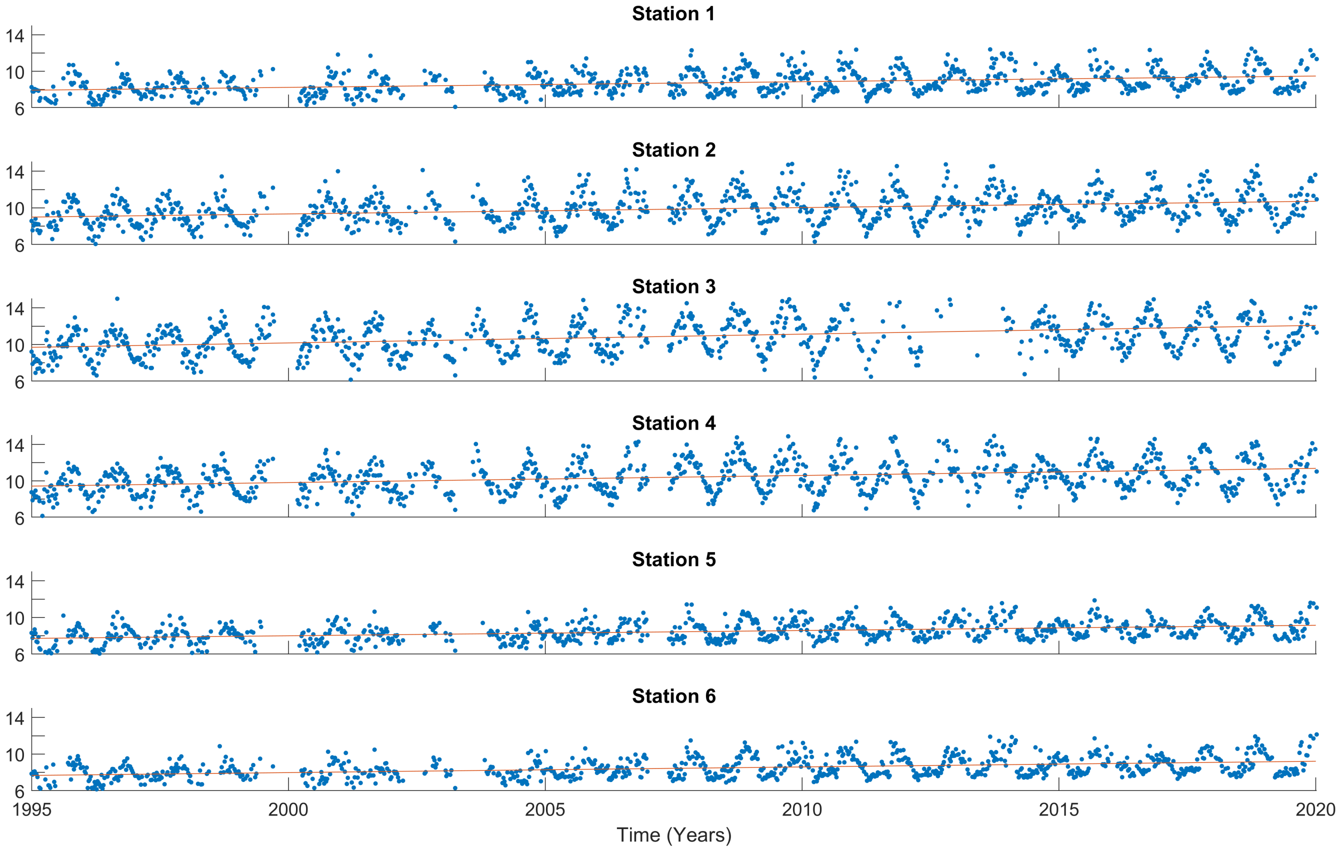

Figure S3. The [H^+] time series (blue dots) within the 5-10 m range at the 6 stations, with a trend indicated by a red line. The y-axis represents [H^+] in nmol kg^{-1}, and the x-axis represents the years.

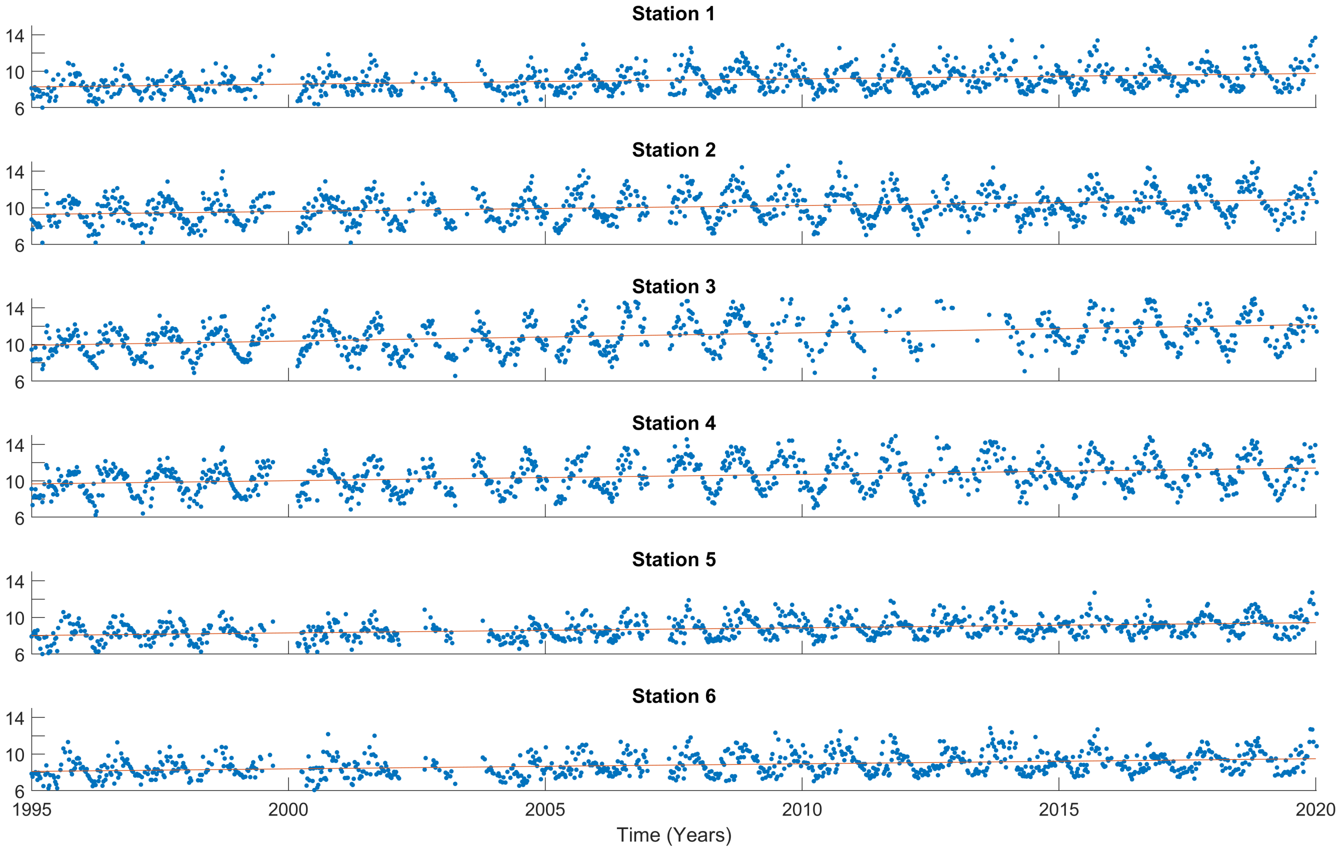

Figure S4. The [H^+] time series (blue dots) within the 10-15 m range at the 6 stations, with a trend indicated by a red line. The y-axis represents [H^+] in nmol kg^{-1}, and the x-axis represents the years.

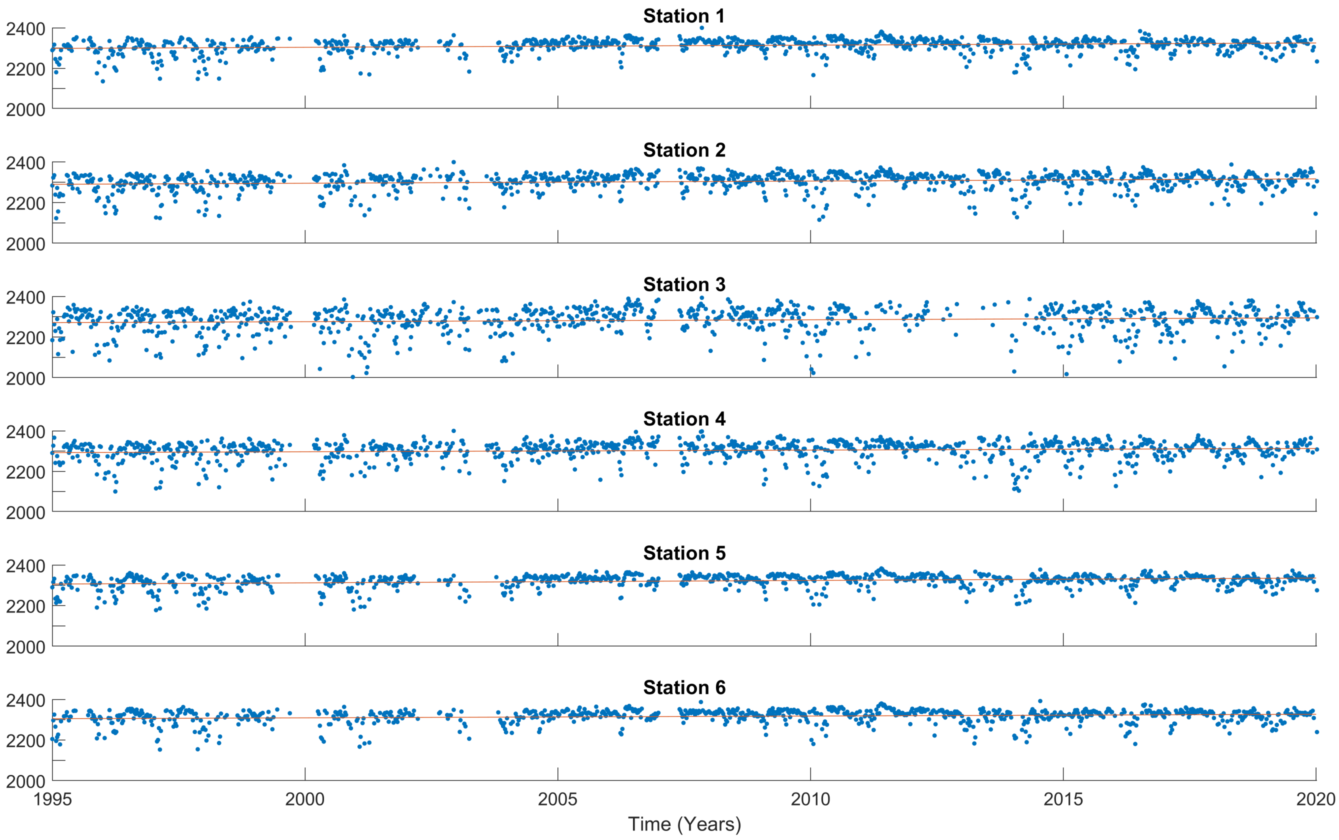

Figure S5. The TA time series (blue dots) are displayed for the 5-10 m range at the 6 stations. The trend is depicted by a red line. The y-axis represents TA in μmol kg^{-1}, and the x-axis represents the years.

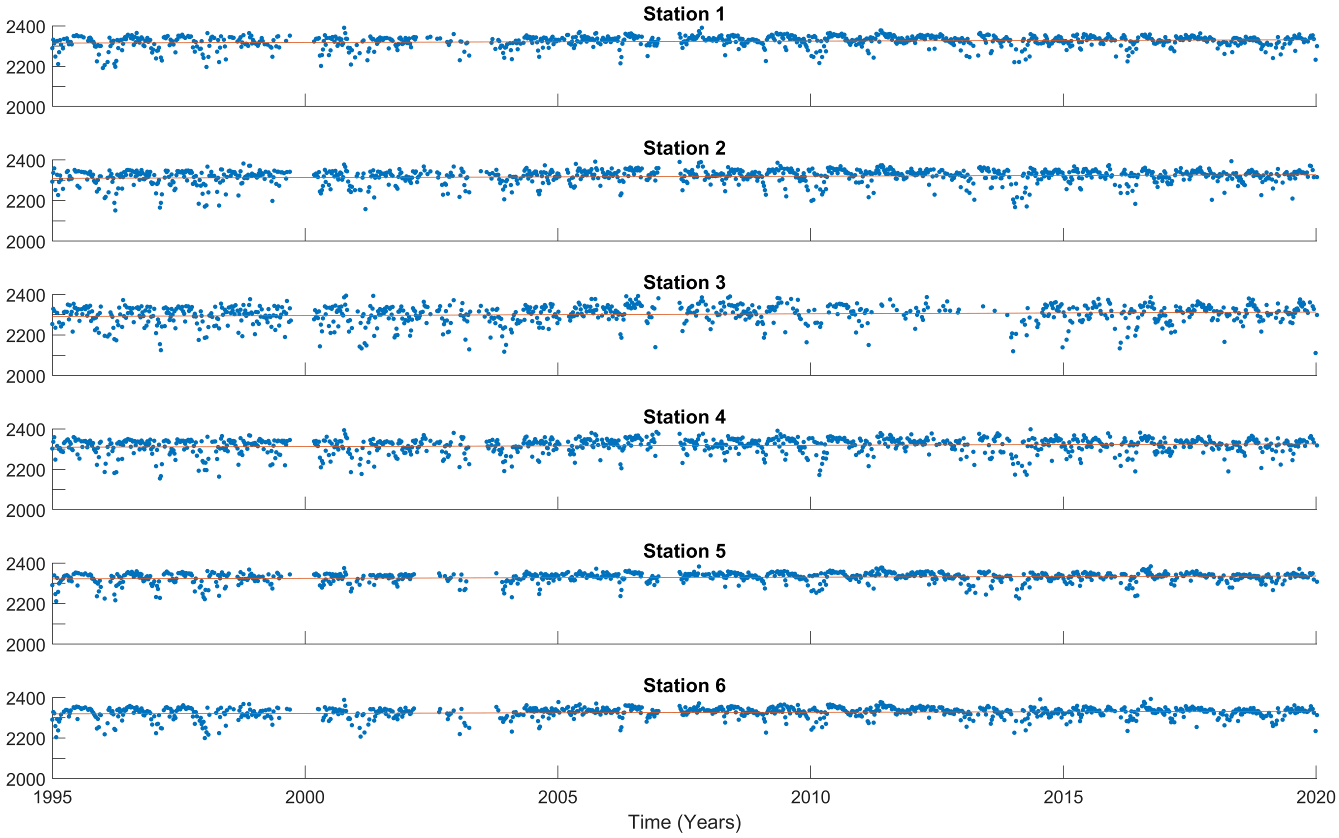

Figure S6. The TA time series (blue dots) are displayed for the 10-15 m range at the 6 stations. The trend is depicted by a red line. The y-axis represents TA in μmol kg^{-1}, and the x-axis represents the years.
